# Therapeutic efficacy of a novel βIII/βIV-tubulin inhibitor (VERU-111) in pancreatic cancer

**DOI:** 10.1186/s13046-018-1009-7

**Published:** 2019-01-23

**Authors:** Vivek K. Kashyap, Qinghui Wang, Saini Setua, Prashanth K. B. Nagesh, Neeraj Chauhan, Sonam Kumari, Pallabita Chowdhury, Duane D. Miller, Murali M. Yallapu, Wei Li, Meena Jaggi, Bilal Bin Hafeez, Subhash C. Chauhan

**Affiliations:** Department of Pharmaceutical Sciences, Institute of Biomarker and Molecular Therapeutics (IBMT), College of Pharmacy, University of Tennessee Health Science Center, 881 Madison Avenue, Memphis, TN 38163 USA

**Keywords:** VERU-111, Pancreatic cancer, β –tubulins, miR-200c, βIII/βIV-tubulin inhibitor

## Abstract

**Background:**

The management of pancreatic cancer (PanCa) is exceptionally difficult due to poor response to available therapeutic modalities. Tubulins play a major role in cell dynamics, thus are important molecular targets for cancer therapy. Among various tubulins, βIII and βIV-tubulin isoforms have been primarily implicated in PanCa progression, metastasis and chemo-resistance. However, specific inhibitors of these isoforms that have potent anti-cancer activity with low toxicity are not readily available.

**Methods:**

We determined anti-cancer molecular mechanisms and therapeutic efficacy of a novel small molecule inhibitor (VERU-111) using in vitro (MTS, wound healing, Boyden chamber and real-time xCELLigence assays) and in vivo (xenograft studies) models of PanCa. The effects of VERU-111 treatment on the expression of β-tubulin isoforms, apoptosis, cancer markers and microRNAs were determined by Western blot, immunohistochemistry (IHC), confocal microscopy, qRT-PCR and in situ hybridization (ISH) analyses.

**Results:**

We have identified a novel small molecule inhibitor (VERU-111), which preferentially represses clinically important, βIII and βIV tubulin isoforms via restoring the expression of miR-200c. As a result, VERU-111 efficiently inhibited tumorigenic and metastatic characteristics of PanCa cells. VERU-111 arrested the cell cycle in the G2/M phase and induced apoptosis in PanCa cell lines via modulation of cell cycle regulatory (Cdc2, Cdc25c, and Cyclin B1) and apoptosis - associated (Bax, Bad, Bcl-2, and Bcl-xl) proteins. VERU-111 treatment also inhibited tumor growth (*P* < 0.01) in a PanCa xenograft mouse model.

**Conclusions:**

This study has identified an inhibitor of βIII/βIV tubulins, which appears to have excellent potential as monotherapy or in combination with conventional therapeutic regimens for PanCa treatment.

**Electronic supplementary material:**

The online version of this article (10.1186/s13046-018-1009-7) contains supplementary material, which is available to authorized users.

## Background

Pancreatic cancer (PanCa) is one of the most lethal cancers and ranked as the fourth most common cause of cancer-related deaths among both men and women in the United States [1]. The management of PanCa is exceptionally difficult due to poor response to available therapeutic regimens [2]. Thus, the identification of newer, highly effective therapeutic agents with no or minimal toxicity is highly desirable for the improved management of PanCa.

Tubulins associated cellular structures composed of α–β tubulin heterodimers are critically involved in a wide range of cellular processes, including maintenance of cell shape, cell motility and cell division, thus are considered important molecular targets in cancer therapeutics [3, 4]. Among these, β-tubulins (namely βI, βIIa, βIIb, βIII, βIVa, βIVb, βV and βVI) isotypes are reported to have higher expression in several cancer types such as lung, breast, prostate, gastric and melanoma [5–10]. Moreover, increased expression of β-tubulins is correlated with disease progression, overall survival and resistance to chemotherapeutic agents in different cancers [10, 11]. The aberrant expression of the β-tubulins (such as βIVa and βIVb) increases in metastatic cancer cells [12, 13]. Recent studies have reported major roles of βIII and βIV-tubulins in PanCa as these isotypes are highly expressed in pancreatic tumors, while absent in normal pancreas (acinar and pancreatic islets) [14, 15]. The expression of these tubulins has been associated with PanCa progression, metastasis and chemoresistance [13, 16–20]. Additionally, βIII-tubulin knockdown reduced the pancreatic tumor growth and metastasis in an orthotropic xenograft mouse model [15]. These studies suggest a major role of βIII and βIV-tubulins in PanCa, thus can be precise and logical molecular targets for PanCa treatment. Although numerous tubulin inhibitors have been reported, nevertheless, selective inhibitors of these specific tubulins are rarely identified [21]. In this study, we have shown that a novel synthetic molecule that is known to overcome multidrug resistance preferentially inhibits the expression of βIII and βIV-tubulins via restoring the expression of miR-200c in PanCa cells [22–24]. For the first time, we have also demonstrated that VERU-111 effectively inhibits growth and metastatic phenotypes of PanCa cells in in vitro and in vivo model systems. Our study suggests potential implications of this novel small molecule in the treatment of PanCa in future.

## Methods

### Synthesis of VERU-111

Synthesis of VERU-111 was carried out as described in our earlier study [24]. Its synthesis scheme and characterization procedures are shown in Additional file 1: Figure S1.

### Antibodies and reagents

Specific monoclonal and polyclonal antibodies of βI-tubulin (cat. # SC- 5274), βIII-tubulin (cat. # SC -51,670) were obtained from Santa Cruz Biotechnology. βV-tubulin (cat. # ab110592) was obtained from Abcam. βIIb-tubulin (cat. # PA5–60448), βIVb-tubulin (cat. # PA5–25050), βVI-tubulin (cat. # PA5–21826) were obtained from Invitrogen. Antibody βIIa - tubulin (cat. # TA345669) and βIVa-tubulin (cat. # TA-340088) were obtained from OriGene. Cyclin B1 (cat. # 4138), Cdc25C (cat. # 4688), Cdc2 (cat. # 77055), p-Cdc2Tyr15 (cat. # 9111), Bax (cat. # 2772), Bcl-2 (cat. # 2876), Bad (cat. # 9292), Bcl-xL (cat. # 2762), Caspase 3 (cat. # 9665), Cleaved caspase 3 (cat. # 9661), Caspase 9 (cat. # 9508) and PARP (cat. # 9542) were purchased from Cell Signaling Technology. The anti-mouse IgG HRP and rabbit IgG HRP-linked secondary antibodies were procured from Promega (Madison, WI). The hematoxylin stain was purchased from Fisher Scientific and the Annexin V/FITC apoptosis kit from Bio-Rad (Hercules, CA). MTT(3-(4,5-dimethyl-2-thia-zolyl)-2,5-diphenyl-2-H-tetrazoliumbromide), Phenylmethanesulfonyl fluoride (PMSF), fetal bovine serum (FBS), eukaryotic protease inhibitor cocktail, pyruvic acid and Propidium iodide (PI), were purchased from Sigma–Aldrich Co. (St. Louis, MO) or Fisher Scientific (Pittsburgh, PA). Pan Caspases inhibitor (Z-VAD-FMK cat. FMK001) was obtained from R&D systems, USA.

### Cell lines

Panc-1, AsPC-1, and HPAF-II cells were obtained from ATCC and cultured in their respective media as DMEM, RPMI-1640 and DMEM/F12 containing 10% FBS and 1% antibiotic/antimycotic. Cells were maintained in CO_2_ incubator at 37 °C with 98% humidity and 5% CO_2_ environment.

### Cell proliferation and colony forming assays

The anti-proliferative effect of VERU-111, colchicine, paclitaxel and vinorelbine on PanCa cells was examined by MTT and colony formation assays as described in our previous studies [25].

### miR transfection and qRT-PCR

Cells were transfected using Lipofectamine 2000 (Invitrogen) following the manufacturer’s protocol. Briefly, Panc-1 and AsPC-1 cells were transiently transfected with miR-200c mimics or non-targeting control mimic (NC) at 100 nM (Applied Biosystems). Total RNA was extracted from control and VERU-111 treated PanCa cells using TRIzol™ reagent (Invitrogen, Life Technologies, Grand Island, NY). cDNAs were prepared by SYBR Green RNA Reverse Transcription kit. The mRNA expression of beta tubulin isotypes were analyzed by qPCR using specific primers sequences as described earlier in Additional file 2: Table S1 [26]. For miRNA detection, 100 ng total RNA was reverse transcribed into cDNA using specific primers designed for miRNA analysis (Applied Biosystems, Foster City, CA). Expression of miRNA 200c was determined by qPCR using the Taqman PCR master mix and specific primers designed for the detection of mi-R200c (Applied Biosystems). The expression of miR-200c was normalized with endogenous control RUN6B [27].

### Western blot and confocal microscopy analyses

PanCa cells (1 × 10^6^) were treated with VERU-111, colchicine, and vinorelbine (5–20 nM) for 24 h. Total cell lysates were processed for Western blot analysis for detecting protein levels of various beta tubulin isoforms and other oncoproteins [28, 29]. Confocal immunofluorescence microscopy was performed for microtubule subcellular localization [28]. Briefly, PanCa cells were seeded on glass coverslips in 6 well plates and incubated overnight. Cells were treated with VERU-111and tubulins targeting agent, fixed and incubated primary antibodies overnight. This was followed by incubation with or Alexa Fluor 488, donkey secondary antibodies for 1 h. The images were then captured with a Zeiss 710 Confocal microscope and Zen imaging software (Zeiss). To investigate whether caspase 3 and 9 are involve in VERU-111 induce apoptosis, PanCa cells were treated with Z-VAD-FMK (20 μ) for 2 h followed by VERU-111 for 24 h. The protein levels of cleaved and pro-Caspase-3, 9, and PARP protein was analyzed by Western blot analysis.

### In situ hybridization (ISH)

To determine the expression of miR-200c, we performed in situ hybridization assays in excised tumor tissues of control and VERU-111 treated mice by Biochain kit (Biochain, San Francisco, CA) as described [28].

### Cell migration and invasion assays

Cell migration assay was performed in Corning’s 96-well HTS Transwell as per manufacturer’s instructions. Cells were treated with VERU-111 (1.25–10 nM) for 24 h, fixed with 4% para-formaldehyde, and with stained with crystal violet. Further, a wound healing assay was also performed to evaluate the effect of VERU-111 on cell migration. Images of the wounds were monitored under a phase-contrast microscope at 10X magnification. For Invasion assays, cells were grown on BD Biocoat Matrigel Invasion Chambers (BD Biosciences, Heidelberg, Germany) according to the manufacturer’s protocol. Cells were then treated with different concentrations of VERU-111 and incubated for 24 h. Invaded cells were fixed, stained and counted as described in our previous study [30].

### Real time cell proliferation, migration and invasion by xCELLigence assays

The effect of VERU-111 on proliferation, migration and invasion of Panc-1 and AsPC-1 cells was investigated by real time xCELLigence technology as described in our previous study [31]. PanCa cells were seeded per chamber for cell proliferation, migration, and invasion assays in E plates following the xCELLigence real time cell analyzer manuals. VERU-111 and vehicle control were added at indicated time and concentrations. The baseline cell index for VERU-111 treated cells compared to control cells was calculated for at least two measurements from three independent experiments.

### Cell cycle analysis

Effect of VERU-111 on cell cycle arrest of PanCa (Panc-1 and AsPC-1) cells was analyzed by flow cytometric analysis. Briefly, approximately 70% confluent cells were synchronized by overnight incubation in FBS free media. Cells were treated with VERU-111 (0, 5, 10, and 20 nM) for 24 h, harvested, fixed overnight in ice-cold ethanol (70%) followed by incubation with RNAse and incubated with the DNA staining Propidium iodide (Sigma) for flow cytometry. Data regarding the number of cells in different phases of the cell cycle was analyzed by BD Accuri C6; Becton-Dickinson, Mountain View, CA.

### Apoptosis assay

The effect of VERU-111 on apoptosis induction in PanCa cells was analyzed by Annexin V-7AAD staining and mitochondrial membrane potential (ΔΨm). Briefly, PanCa cells (1 × 10^6^) were treated with VERU-111 (5–40 nM) for 24 h. To investigate whether caspase 3 and 9 are involve in VERU-111 induce apoptosis, PanCa cells were treated with Z-VAD-FMK (20 μM) for 2 h followed by VERU-111 for 24 h. Cells were then collected and stained with Annexin V and 7-AAD (5 μl/100 μl of cell suspension) and analyzed by Accuri C6 Flow Cytometer setting FL2 and FL3 channels. Effect of VERU-111 on mitochondrial membrane potential (ΔΨm) in PanCa cells was analyzed by uptake of tetramethylrhodamine (TMRE) staining. Briefly, PanCa cells were treated with VERU-111 (5–20 nM) for 24 h, and further incubated with TMRE (100 nM) for 20 min; Fluorescence intensities of TMRE stained cells were measured by flow cytometry.

### Tumor xenograft study

To determine effective tumor growth inhibitory dose of VERU-111 at tumor site, we performed ectopic xenograft studies in athymic nude mice. For that, six-week-old female athymic nude mice (nu/nu) were purchased from Jackson laboratory and maintained in a pathogen-free environment. All procedures were carried out as per the approved UTHSC Institutional Animal Care and Use Committee (UTHSC-IACUC) protocol. To establish ectopic xenograft tumors in mice, AsPC-1 cells (2 × 10^**6**^ cells) were suspended in phosphate buffer saline (PBS) and Matrigel (BD Biosciences) solution (1:1 ratio) and then injected subcutaneously on the dorsal flanks of each mouse. Tumor growth in mice was monitored using a digital Vernier caliper. When tumor volume reached ~ 200 mm^3^, mice were divided into control (*n* = 6) and VERU-111 (*n* = 6) treatment groups. Mice were administered with VERU-111 (50 μg/mouse/week for 3 weeks; intra-tumorally) or vehicle control (PBS). Tumor volumes were measured weekly and calculated by the formula 0.5238 x L x W x H, where L is length, W is width and H is the height of the tumor. Mice were euthanized when tumor volume of control mice reached ~ 1000 mm^3^. At the time of sacrifice, tumors were excised and processed for RNA, tissue lysates, histopathology and slides preparation (5 μm section).

### Immunohistochemistry

The effect of VERU-111 was determined on the expression of PCNA and tubulin isoforms in excised tumors by immunohistochemistry using Biocare kits (Biocare Medical, Concord, CA) as described previously [28].

### Statistical analysis

The data discussed above are presented in terms of mean values and the SEM of several independent experiments. *p*-values < 0.05 were considered statistically significant. All statistical analyses were performed using the Statistical Package for the Social Sciences, version 11.5 (SPSS Inc., Chicago, IL).

## Results

### VERU-111 inhibits growth and clonogenic potential of PanCa cells

**Fig. 1:**
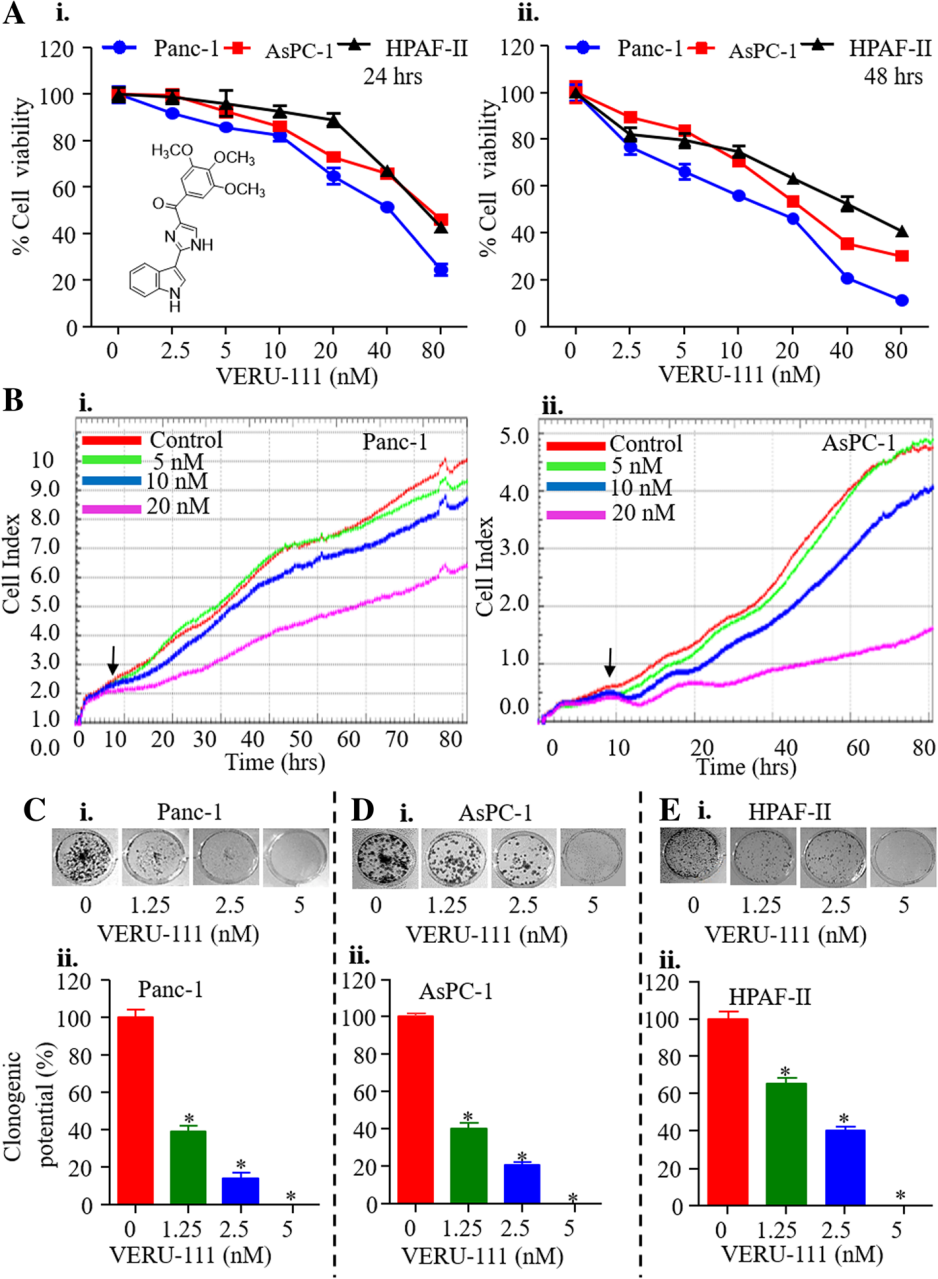
VERU-111 inhibits growth characteristics of PanCa cells. **(A)** Effect of VERU-111 on viability of Panc-1, AsPC-1, and HPAF-II cells. Structure of VERU-111 (2-(1H-indol-3-yl)-1H-imidazol-4-yl) (3, 4, 5-trimethoxyphenyl)) – methanone) is shown as insert. Cells were treated with indicated concentrations of VERU-111 for 24 **(i)** and 48 **(ii)** hrs and cell viability was determined by MTT assay. **(B)** Effect of VERU-111 on PanCa cells in a real time cell proliferation assay. Cells (5000 cells/well) were seeded in E-plate (xCELLigence) and placed into the xCELLigence Real Time Cell Analyzer (RTCA) DP. After 8–10 h, VERU-111 or the vehicle control was added and the experiment was allowed to run for 80 h. Line graphs show the average baseline cell index of control and VERU-111-treated cells. (**C-E**) Effect of VERU-111 on clonogenic potential of PanCa cells as determined by anchorage dependent colony formation assay. Representative images of colony formation assay and bar graphs indicating quantification of cells are shown at indicated concentrations of VERU-111. (Values means ± SEM; *n* = 3). Asterisk (*) denotes the significant value *p* < 0.05

We first assessed the cytotoxic effect of VERU-111 against various human PanCa cell lines (AsPC-1, Panc-1 and HPAF-II). In this experiment, cells were treated with various concentrations of VERU-111 (1.25–160 nM) for 24 and 48 h, and cell viability was determined by MTT assay. VERU-111 inhibited the growth of PanCa cells in a dose and time-dependent manner (Fig. 1Ai-ii). The IC_50_ of VERU-111 was 25, 35 and 35 nM in Panc-1, AsPC-1 and HPAF-II, respectively after 24 h treatment (Fig. 1Ai), while 48 h post-treatment it was 11.8, 15.5, and 25 nM (Fig. 1Aii). We further evaluated growth inhibitory effect of VERU-111 in real time with the help of xCELLigence system. This system monitors cell growth by measuring electrical impedance, which is expressed as a cell index. A growth curve, recorded as the basal cell index value, showed that VERU-111 significantly reduced the cell index in a dose-dependent manner compared to vehicle treated cells (Fig. 1Bi-ii). To determine the long-term effect of VERU-111 on the growth, we performed colony formation assays in PanCa cells. VERU-111 (1.25–5 nM) treatment significantly reduced the number of colonies in a dose-dependent manner (Fig. 1C-E) as compared to respective control groups.

### VERU-111 inhibits mRNA expression and protein stability of β-tubulin isotypes in PanCa cells

**Fig. 2:**
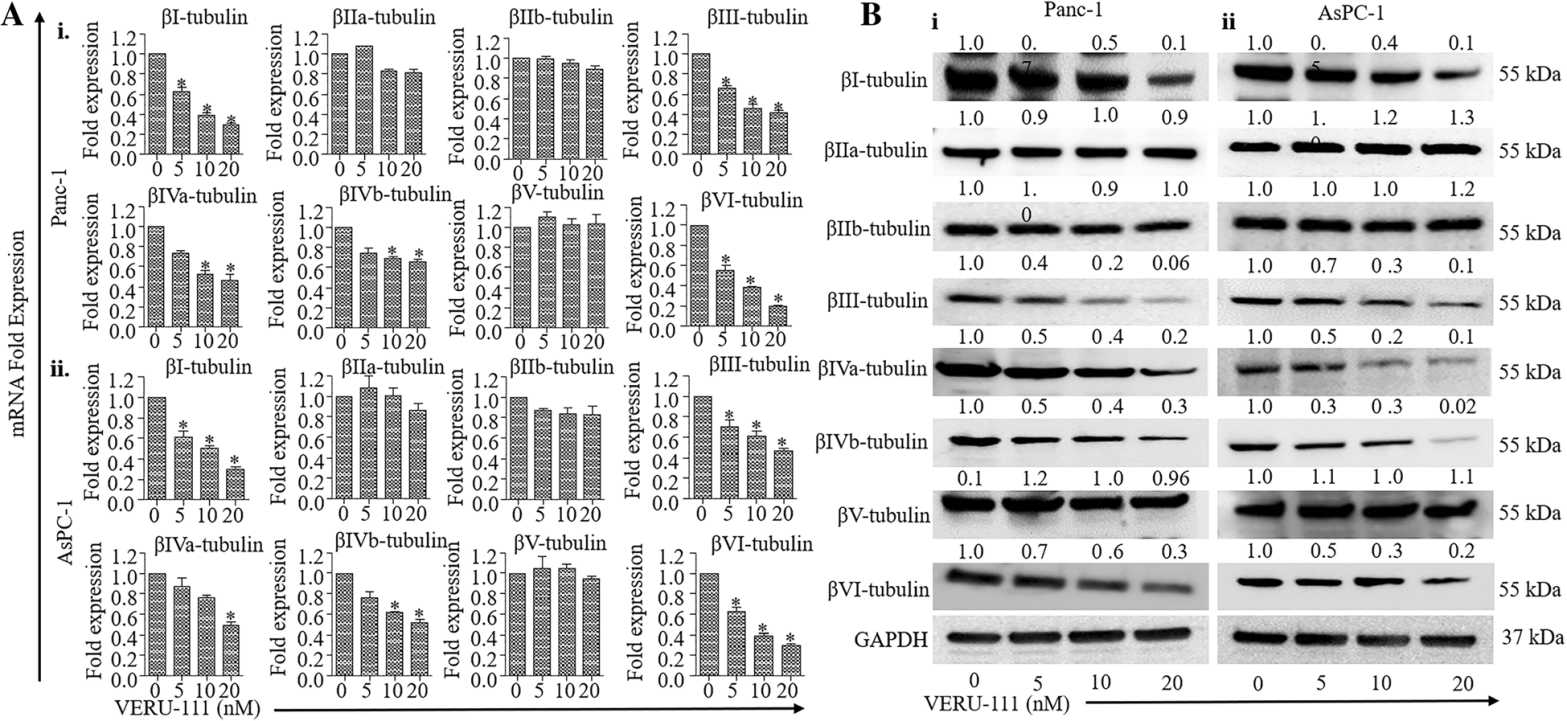
Effect of VERU-111 on the expression of β-tubulin isotypes in PanCa cells. (**A**) Effect of VERU-111 on mRNA expression of βI, βIIa, βIIb, βIII, βIVa, βIVb, βV and βVI-tubulins in Panc-1 (i) and AsPC-1 (ii) cells. Briefly, cells were treated with the indicated concentrations of VERU-111 for 24 h. RNAs were isolated and transcribed for cDNA preparation. qPCR was performed to determine the mRNA expression of indicated tubulin isotypes. GAPDH was used as an internal control. Bar graphs represent relative fold expression of various tubulins mRNA. (Values mean ± SEM; *n* = 3). Asterisk (*) denotes the significant value *p* < 0.05. (**B**) Effect of VERU-111 on protein levels of various β-tubulin isotypes in Panc-1 (i) and AsPC-1(ii) cells. Cells were treated with vehicle or indicated concentrations of VERU-111 for 24 h and cell lysates were subjected for Western blot analysis. Equal loading of protein in each well was confirmed by stripping and re-probing of the blots with GAPD for all isoform were provided in Additional file 7: Figure S2

It is well documented that βIII and βIV-tubulins are involved in pancreatic carcinogenesis and are potential molecular targets for PanCa therapeutics [32]. Thus, we first investigated the effect of VERU-111 on the expression of βIII and βIV-tubulins in PanCa cells. VERU-111 (5–20 nM) treatment significantly (*p* < 0.01) inhibited the mRNA expression of βIII and βIV-tubulins, in PanCa cells (Fig. 2A) as determined by qRT-PCR. Western blot analysis results also demonstrated VERU-111 mediated inhibition of the expression of βIII and βIV-tubulins (Fig. 2B). We also evaluated the effect of VERU-111 on other tubulin isotypes to determine its specificity at both the mRNA and protein level. We observed differential inhibition in the expression of βI and βVI tubulins protein and mRNA levels (Fig. 2A-B). However, the expression of βIIa, βIIb and βV-tubulins remain unaffected in both the tested cell lines (Fig. 2A-B).

### VERU-111 inhibits βIII-tubulin and causes disruption of microtubular dynamics in PanCa cells

**Fig. 3:**
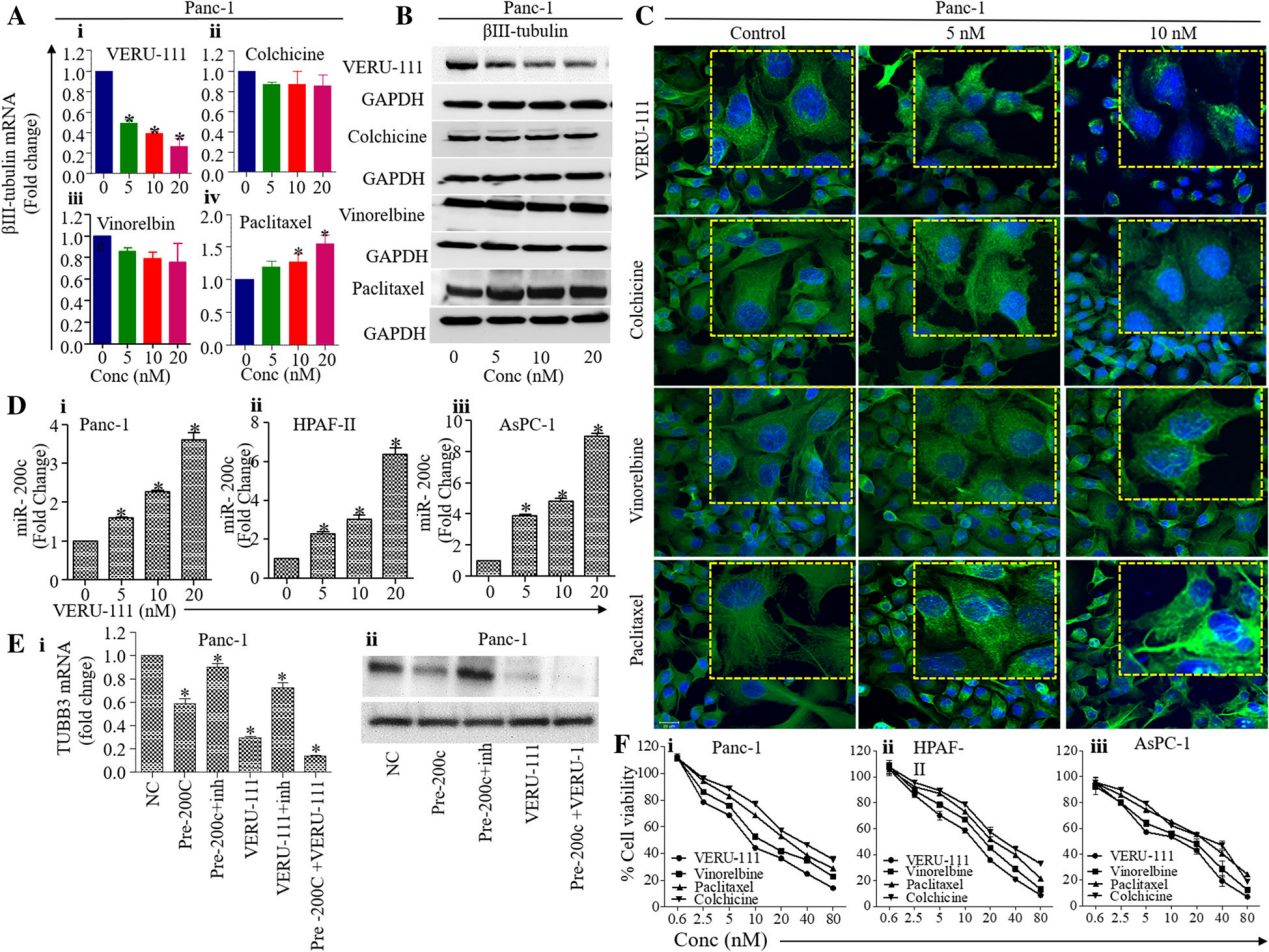
VERU-111 repressed the expression of βIII-tubulin and restored miR-200c expression in PanCa cells. (**A**) Effect of VERU-111 (i), colchicine (ii), vinorelbine (iii) and paclitaxel (iv) treatment on the mRNA expression of βIII-tubulin in PanCa cells as determined by qPCR analysis. Bar graphs represent fold change mRNA expression of βIII-tubulin compared to control group. (Values means ±SEM; *n* = 3). *p* < 0.05. (**B**) Western blot analysis results indicating the effect of VERU-111, colchicine and vinorelbine on β-tubulin III in Panc-1 cells at 24 h post-treatment. **(C)** PanCa cells (Panc-1) were treated with control (vehicle) or VERU-111, colchicine, vinorelbine and paclitaxel at 5–10 nM for 18 h. These cells were processed for immunofluorescence analysis using anti- βIII-tubulin antibody (green) and DAPI (blue). The images were captured with a Zeiss 710 Confocal microscope and Zen imaging software (Zeiss) at × 63 magnifications. **(D)** Effect of VERU-111 on the expression of miR-200c in Panc-1 (i), AsPC-1 (ii) and HPAF-II (iii) cells as determined by qPCR analysis. RNU6B was used as an internal control. (**E**) Effect of VERU-111 on the expression of βIII-tubulin in miR-200c mimic or inhibitor transfected Panc-1 cells as determined by qPCR (i) and WB analysis (ii). Cells were transfected with 100 nM of miR-200c mimic (pre-200c) or miR-200c inhibitor or scrambled miRNA (negative control) for 48 h followed by VERU-111 (20 nM) treatment for 24 h. RNA was isolated and transcribed for cDNA and mRNA expression of βIII-tubulin was determined by qPCR (i). Data in bar graph indicate fold change mRNA expression of βIII-tubulin. (Values mean ± SEM; *n* = 3). Asterisk (*) denote the significant value *p <* 0.05. In a same parallel experiment, protein lysates were prepared and subjected for Western blot analysis to determine the protein levels of βIII-tubulin. Equal loading of protein was determined by stripping and probing the blot with GAPDH antibody. **(F)** Comparative effect of VERU-111, colchicine and vinorelbine, on cell viability of Panc-1 (i), AsPC-1 (ii), and HPAF-II (iii) cells as determined by MTT assay. Line bar graphs indicate percent cell viability compared to control group in response to VERU-111, colchicine, vinorelbine, and paclitaxel treatment after 48 h treatment. Values in graph represent mean ± SEM of three independent experiments. Asterisk (*) denotes the significant value *p* < 0.05

Next, we compared the effect of VERU-111 on the expression of βIII-tubulin with known microtubule targeting agent’s colchicine and vinorelbine (destabilizing agent) and paclitaxel (microtubule stabilizing). In this experiment, Panc-1 cells were treated with 5–20 nM of VERU-111, colchicine, vinorelbine and paclitaxel for 24 h, and RNA and protein lysates were prepared to determine the mRNA expression and protein level of βIII-tubulin. VERU-111 effectively inhibited mRNA expression (Fig. 3Ai) and protein levels (Fig. 3B) of βIII-tubulin. Destabilizing agents (colchicine and vinorelbine) did not show significant effect on the expression of βIII-tubulin (Fig. 3Aii-iii and Fig. 3B). Paclitaxel treatment, however, stabilized the expression of βIII-tubulin (Fig. 3Aiv and Fig. 3B). To determine effects of VERU-111 on microtubule networks and cell morphology, we utilized confocal microscopy. For that, Panc-1 cells were treated with VERU-111, colchicine, vinorelbine and Paclitaxel at different concentrations for 18 h. The untreated cells appeared typical nuclear and cytoskeleton structures, extending throughout the cell to the cell periphery while, VERU-111 treated cells show reduced and fragmented βIII-tubulin microtubule network (Fig. 3C). Colchicine and vinorelbine did not affect the morphological changes in microtubule. Paclitaxel treatment led the formation of shortened but unusually highly defined microtubules bundles concentrated toward the nucleus (Fig. 3C). The microtubule fragmentation with disruption observed by VERU-111 is consistent with its putative mechanism of action (i.e., inhibiting tubulin polymerization).

### VERU-111 restores the expression of miR-200c via targeting βIII-tubulin

It has been reported that miR-200c directly targets βIII-tubulin in PanCa cells. Thus, we sought to determine the effect of VERU-111 on the expression of miR-200c [33]. Interestingly, VERU-111 treatment induced the expression of miR-200c in Panc-1, AsPC-1 and HPAF-II cells, when compared with control treated cells (Fig. 3D). We next determined if inhibition of miR-200c minimizes the effect of VERU-111 on the expression of βIII tubulin. For that, cells were transfected with miR-200c mimics in Panc-1 cells that resulted to the inhibition of βIII-tubulin expression of, which was rescued with the transfection of miR-200c inhibitor (Fig. 3Ei-ii). A combination of VERU-111 and miR-200c mimic treatment showed a pronounced effect on the inhibition of βIII tubulin expression (Fig. 3E). These results suggest that VERU-111 inhibits βIII tubulin expression via restoration of the expression of miR-200c in PanCa cells. Next, we performed comparative anti-proliferative effect of VERU-111, colchicine, vinorelbine and Paclitaxel in PanCa (Panc-1, AsPC-1 and HPAF-II) cells using MTT assays. Interestingly, VERU-111 showed the most potent anti-proliferative activity compared to other agents (Fig. 3F).

### VERU-111 inhibits migration and invasive potential of PanCa cells

**Fig. 4:**
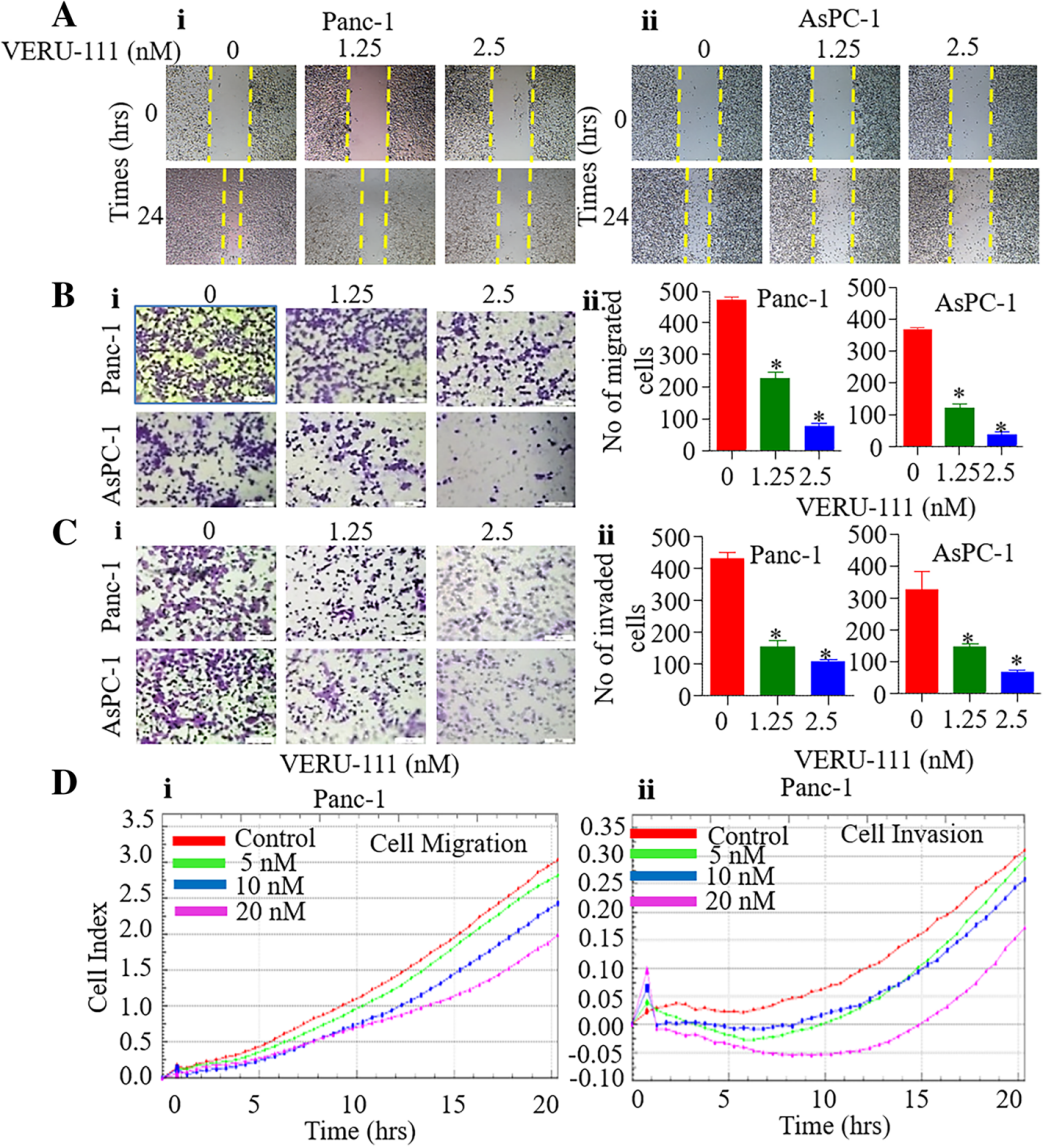
Effect of VERU-111 on invasion and migration of PanCa cells. (**A**) Effect of VERU-111 on migration of Panc-1 and AsPC-1 cells determine by wound healing assays. The wounded monolayer was incubated in different concentrations of VERU-111 for 24 h. Images of wound healing assays (magnification, × 10) of Panc-1 (i) and AsPC-1 (ii) cells of control and VERU-111 treatment groups after 24 h. (**B**) Effect of VERU-111 on migration of AsPC-1 and Panc-1 cells using 96-transwell chamber plate. Representative images of migratory Panc-1 and AsPC-1 cells (i) of control and VERU-111 treatment groups after 24 h. Bar graphs (ii) indicating number of migratory Panc-1 and AsPC-1 cells in control and VERU-111 treatment groups. (**C**) Effect of VERU-111 on invasion of Panc-1 and AsPC-1 cells (i) as determined by Boyden’s Chamber assay. Representative images of control and VERU-111 treatment groups were captured at 20x magnification after 24 h. Bar graphs (ii) indicate number of invaded Panc-1 and AsPC-1 cells. **(D)** Effect of VERU-111 on real time migration (i) and invasion (ii) of Panc-1 cells using *xCELLigence assay*. Results are presented as means ± SEM (*n* = 3). Asterisk (*) denotes the significant value *p* < 0.05

In addition to anti-proliferative activity, we elucidated anti-metastatic potential of VERU-111 by determining its effect on the invasive and migratory characteristics of PanCa cells. To address this, we first performed wound healing assays to determine the effect of VERU-111 on the migration of PanCa cells. Our results revealed remarkable inhibition in migration of both Panc-1 (Fig. 4Ai), AsPC-1 (Fig. 4Aii) and HPAF-II (Additional file 3: Figure S3A) cells when treated with sub-lethal concentrations of VERU-111 (1.25 and 2.5 nM). We further evaluated the effect of VERU-111 on PanCa cell migration by transwell assay. VERU-111 (1.25–2.5 nM) also showed significant (*p* < 0.01) inhibition of Panc-1, AsPC-1 (Fig. 4Bi-ii) and HPAF-II (Additional file 3: Figure S3Bi-ii) cell migration in a dose-dependent manner. VERU-111 at sub lethal concentrations (1.25–2.5 nM) also significantly (*P* < 0.01) inhibited invasion of Panc-1, AsPC-1 (Fig. 4Ci-ii) and HPAF-II (Additional file 3: Figure S3 Ci-ii) cells as compared to the vehicle treatment group. The effect of VERU-111 on migration and invasion of PanCa cells was further confirmed using the xCELLigence system. VERU-111 also dose-dependently (5–20 nM) reduced the baseline cell index of PanCa cells as compared to control, which reflects potent inhibitory effects of VERU-111 on PanCa cells migration (Fig. 4Di) and invasion (Fig. 4Dii).

### VERU-111 arrests cell cycle in G2/M phase and induces apoptosis in PanCa cells

**Fig. 5:**
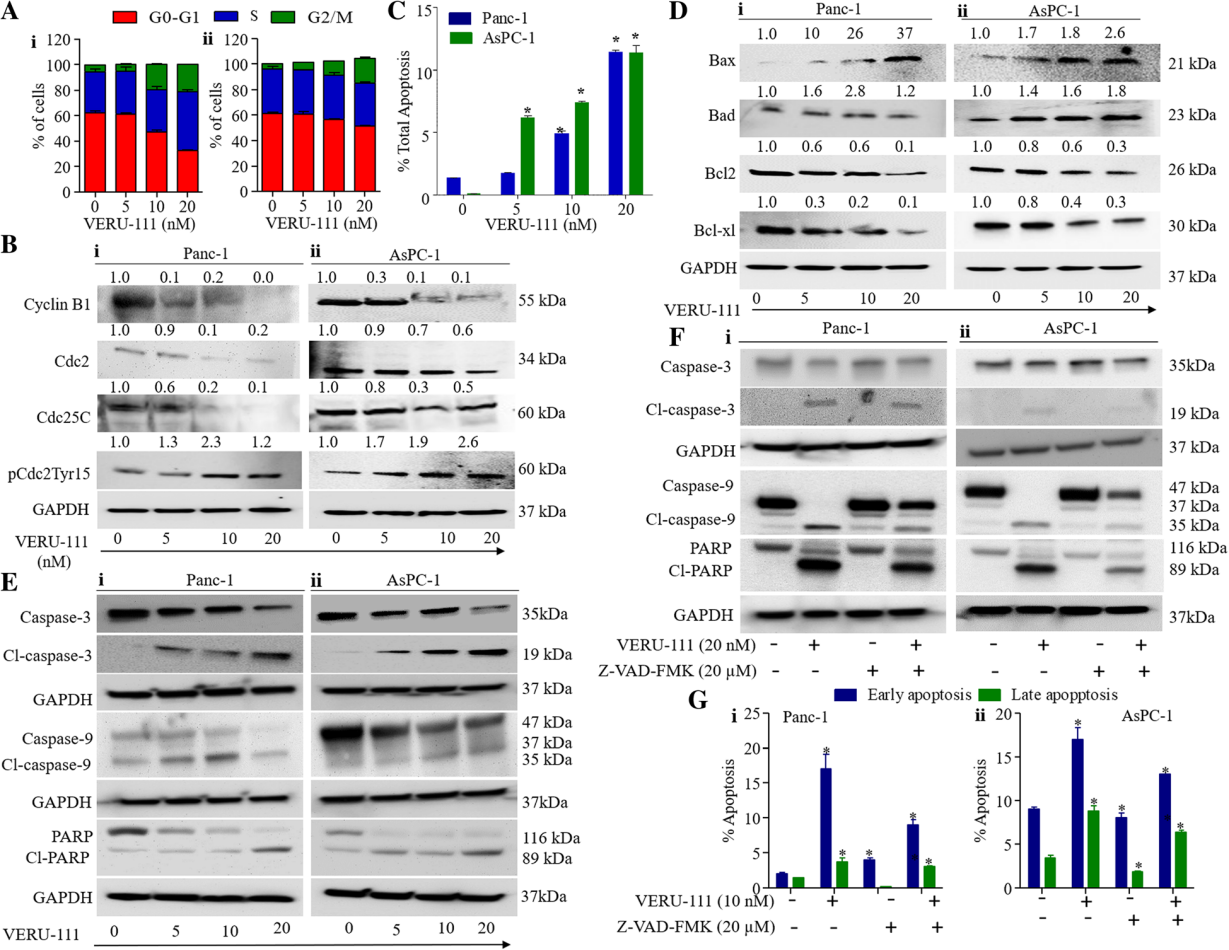
Effect of VERU-111 on cell cycle distribution and apoptosis in PanCa cells. **(A)** Effect of VERU-111 on cell cycle distribution of Panc-1 and AsPC-1 cells. Briefly, Cells were treated with VERU-111 for 24 h and analyzed by flow cytometric analysis using Propidium Iodide. Bar graph showing cell cycle distribution at different phases in G0-G1, S, and G2M as analyzed by MOTFIT Analysis Software (i-ii). **(B)** Effect of VERU-111 on protein levels of cell cycle regulatory proteins (Cyclin B1, Cdc25c, Cdc2, and pCdc2Tyr15) in Panc-1(i) and AsPC-1(ii) cells as determined by Western blot analysis. **(C)** Effect of VERU-111 on apoptosis induction. Briefly, cells were treated with indicated concentrations of VERU-111 for 24 h and apoptosis induction was analyzed by flow cytometry using Annexin V-7AAD Apoptosis kit. Bar graphs showing dose-dependent increase of apoptotic cells in VERU-111 treatment. **(D)** Effect of Protein levels of Bax, Bcl-2, Bad and Bcl-xl in Panc-1(i) and AsPC-1(ii) cells in response to VERU-111 treatment for 24 h as determined by Western blot analysis. **(E)** Effect of VERU-111 on protein levels of pro-caspase-3 and 9, cleaved caspase-3 and 9 and PARP cleavage in Panc-1(i) and AsPC-1(ii) cells as determined by Western blot analysis. **(F)** Effect of VERU-111 alone or in combination with caspase inhibitor Z-VAD-FMK on protein level of pro-caspase-3 and 9, cleaved caspase-3 and 9 and PARP cleavage in Panc-1(i) and AsPC-1(ii) cells as determined by Western blot analysis. (**G**) Effect of VERU-111 alone or in combination with caspase inhibitor Z-VAD-FMK on apoptosis induction analyzed by flow cytometry using Annexin V-7AAD Apoptosis kit. Data was acquired by using the Bio-RAD ZE5/Evererst Software v2.1 and analyzed using FlowJo v.10.3**.** Asterisk (*) denotes the significant value *p* < 0.05

It has been reported that tubulin-destabilizing agents block the cell cycle in the G2/M phase due to microtubule depolymerization and cytoskeleton disruption [34]. This prompted us to evaluate the effect of VERU-111 on PanCa cell cycle distribution using flow cytometry. VERU-111 treatment arrested Panc-1 and AsPC-1 cells in G2/M phase in a dose-dependent manner (Fig. 5Ai-ii) and Additional file 4: Figure S4Ai-ii). The complex formation between cdc2 and cyclin B1 is an important event for cell entry into mitosis [35, 36]. Thus, we determined the effect of VERU-111 on cell cycle regulatory proteins. VERU-111 effectively inhibited the expression of cyclin B1, cdc2 and cdc25c in PanCa (Panc-1 and AsPC-1) cells, while enhancing the phosphorylation of cdc2 (Fig. 5Bi-ii). Since, we observed arrest of cell cycle in G2/M phase, we subsequently investigated the effect of VERU-111 on apoptosis induction in PanCa cells by Annexin V-7AAD staining and mitochondrial membrane potential (ΔΨm) using flow cytometer. As shown in Fig. 5C and (Additional file 5: Figure S5Ai-ii), VERU-111 treatment (5–20 nM) resulted in apoptosis induction in both Panc-1 and AsPC-1 cells. It has been shown that mitochondria plays key role in apoptosis through modulation of intrinsic signaling pathway mechanism and decrease in mitochondrial membrane potential (ΔΨm) has been considered as an early event of apoptosis [37]. Thus, we examined the effect of VERU-111 on ΔΨm in PanCa cells using TMRE staining. VERU-111 illustrated a dose-dependent (5–20 nM) decrease of TMRE staining in PanCa cells (Additional file 5: Figure S5Bi-ii). Additionally, we evaluated the effect of VERU-111 on other mitochondrial pro-apoptotic (Bax and Bad) and anti-apoptotic (Bcl2 and Bcl-xL) proteins. VERU-111 (5–20 nM) induced the expression of Bax and Bad and inhibited the expression of Bcl-2 and Bcl-xl proteins (Fig. 5Di-ii). We also investigated the effect of VERU-111 on pro-caspase-3 and 9, cleaved caspase-3 and 9 and PARP cleavage. VERU-111 treatment showed dose-dependent inhibition of pro-Caspase 3 and 9 and activation of Caspase-3 and 9 in both AsPC-1 and Panc-1 cells (Fig. 5Ei-ii). VERU-111 also showed cleavage of PARP protein in pancreatic cancer cells (Fig. 5Ei-ii). These results clearly indicate that VERU-111 induces apoptosis in pancreatic cancer cells. We further confirmed the involvement of caspase 3 and caspase 9 in VERU-111 induced apoptosis in PanCa cells. Our results illustrated that treatment of general caspase inhibitor Z-VAD-FMK rescued the VERU-111 induced apoptosis of PanCa cells (Fig. 5F-G). Treatment of PanCa cells with VERU-111 (20 nM) showed increase expression of cleaved caspase 3, 9 and PARP proteins which was decreased with the addition of Z-VAD-FMK (Fig. 5Fi-ii). We also observed significant decrease number of apoptotic PanCa cells with the treatment of Z-VAD-FMK when compared to VERU-111 treatment alone (Fig. 5Gi-ii and Additional file 5: Figure S5 Ci-ii). These results clearly suggest the involvement of caspase 3 and 9 in VERU-111 induced apoptosis of PanCa cells.

### VERU-111 effectively inhibits the growth of pancreatic tumors in a xenograft mouse model

**Fig. 6:**
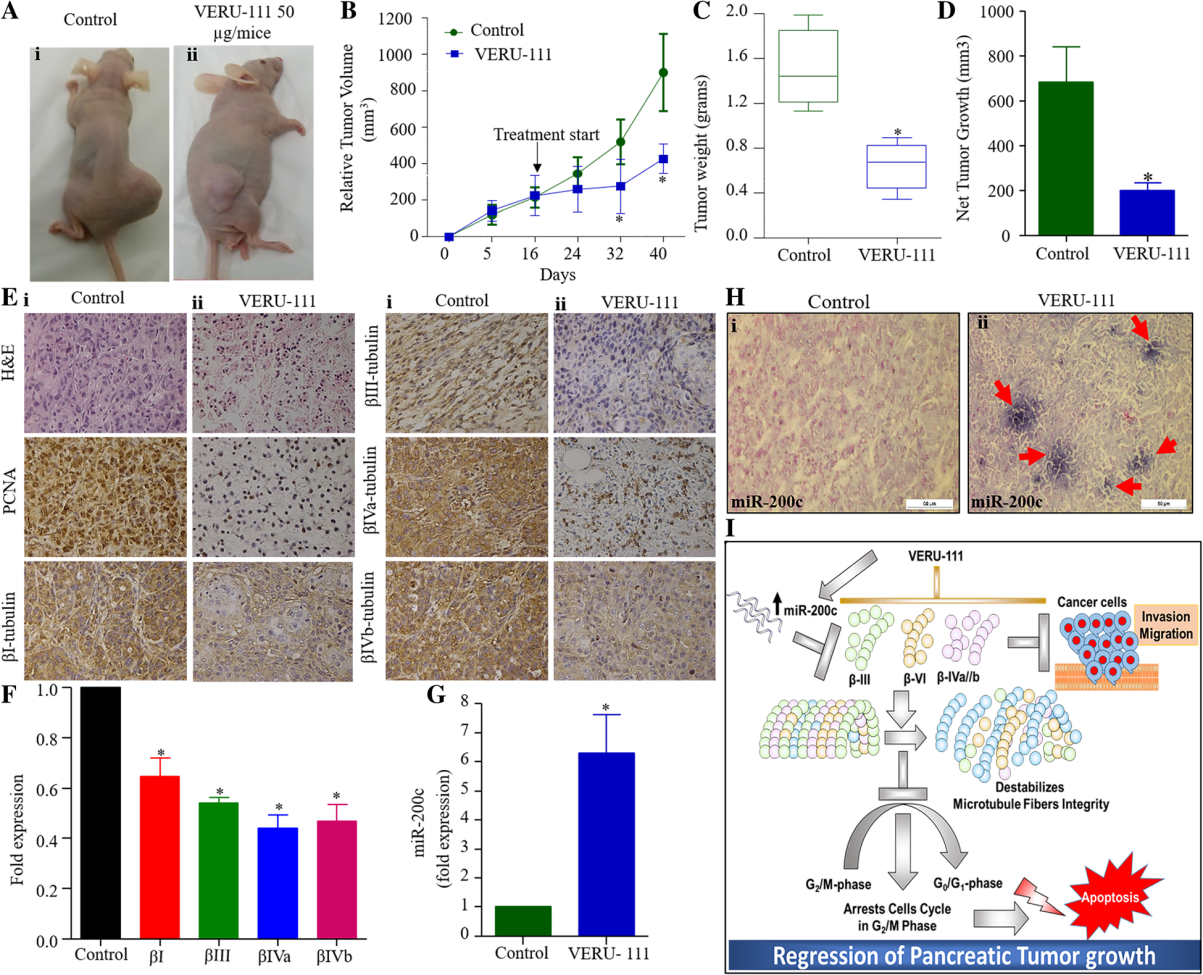
VERU-111 inhibits pancreatic tumor growth. **(A)** Effect of VERU-111 on AsPC-1 cells derived xenograft tumors in athymic nude mice. Representative images of AsPC-1 cells derived xenograft tumor bearing mice of control and VERU-111 treated group. (**B**) Line graph showing average tumor volume of control and VERU-111 treatment group different time points. Data shown in the graph represent mean ± SEM of six tumors of each group. (**C**) Average tumor weight of control and VERU-111 treatment group. (**D**) Net tumor growth of control and VERU-111 treatment group. Data in bar graph represent mean ± SEM of six tumors in each group. Asterisk (*) denotes the significant value *p* < 0.05. (**E**) Representative images of H&E staining of excised xenograft tumors of control (i) and VERU-111 treatment (ii) group. Effect of VERU-111 on the expression of PCNA, βI, βIII, βIVb and βIVb in excised tumor tissues of control (i) and VERU-111 treated (ii) mice as determined by Immunohistochemistry. (**F)** Effect of VERU-111 on mRNA expression of βI, βIII, βIVb and βIVb in excised xenograft tumors of control and VERU-111 treated mice as determined by qPCR. Bar graph represents fold mRNA expression of βI, βIII, βIVb and βIVb (mean ± SEM; *n* = 4). Asterisk (*) denotes the significant value *p* < 0.05. (**G**) Effect of VERU-111 on the expression of miR-200c in excised xenograft tumors of control and VERU-111 treated mice as determined by qPCR (**H**) and representative images of in situ hybridization. (**I**) Proposed model illustrating possible molecular mechanisms of VERU-111 for the inhibition of pancreatic tumor growth. VERU-111 destabilizes microtubule fiber integrality (de-polymerization) via inhibitions of βIII/βIV isotypes, cell cycle arrest and induction of apoptosis. Moreover, VERU-111 also induces miR-200c expression, which negatively regulates β-tubulin III, leading to apoptosis induction and inhibition of invasion/migration of PanCa cells

We next evaluated the therapeutic effect of VERU-111 in a pre-clinical mouse model of PanCa. In this experiment, highly aggressive AsPC-1 cells (2 × 10^6^) were ectopically injected in athymic nude mice to generate xenograft tumors. VERU-111 (50 μg/mice) and its respective vehicle controls (PBS) were administered intra-tumorally once tumor volume reached ~ 200 mm^3^ (3 times per week for 3 weeks). VERU-111 treatment effectively inhibited tumor growth as compared to vehicle-treated group (Fig. 6A-D). The average tumor volume in control mice reached to 900 mm^3^ within 5 weeks, while at this time it was only 400 mm^3^ in VERU-111 treated mice (Fig. 6B). None of the mouse showed any apparent toxicity as we observed constant increase of body weight in VERU-111 treated mice. (Additional file 6: Figure S6). PCNA is one of the markers of cell proliferation, which is upregulated in tumor cells, thus we evaluated if VERU-111 treatment inhibits PCNA expression in tumor tissues. Our IHC analysis demonstrated considerable inhibition of PCNA expression in VERU-111 treated tumor tissues compared to vehicle control treated tumors (Fig. 6E). In accordance with our in vitro data, we also observed the repression of βIII and βIVa and βIVb-tubulins in VERU-111 treated tumors both protein (determined by IHC) and mRNA levels (Fig. 6E, F). Interestingly, VERU-111 treatment also restored the expression of miR-200c in the excised xenograft tumors as determined by qPCR (Fig. 6G) and in situ hybridization (Fig. 6H) assays. In conclusion, we find that VERU-111 disrupts the microtubular dynamics via differentially inhibition of βI, βIII, βIVa, βIVb, and βVI isotypes, G2/M cell cycle arrest, induction of apoptosis and restoration of the miR-200c expression. Overall, these results determined the therapeutic efficacy of a novel tubulin inhibitor (VERU-111) in PanCa models and elucidated its putative cellular and molecular mechanisms (Fig. 6I).

## Discussion

Therapeutic options for pancreatic cancer (PanCa) are very limited, thus it is one of the leading causes of cancer-related mortalities in the United States [38]. Gemcitabine is a Food and Drug Administration (FDA) approved drug for the treatment of local and advanced metastatic PanCa, however its therapeutic efficacy in clinical setting is limited due to emergence of chemoresistance and systemic toxicity [39, 40]. The combination treatment strategy regimen of albumin bound paclitaxel (Abraxane®) along with gemcitabine has led to a 2.5 month increase in overall survival of PanCa patients [41]. It has been shown that Abraxane® increases tumor uptake of gemcitabine via inhibiting gemcitabine metabolizing enzyme (cytidine deaminase) [42]. In spite of these encouraging results, unwanted systemic toxicities of this combination therapy is an unmet clinical challenge.

Tubulin targeting taxanes (such as paclitaxel, docetaxel) have shown great clinical success as anti-cancer agents, which suggest importance of tubulins in cancer therapeutics [43]. However, being Pan-tubulin modulators (stabilizers or inhibitors), these agents suffer from significant systemic toxicities. To address this important issue, investigations are being carried out towards identification of cancer specific molecular targets. Like other cancers, tubulins play a major role in the development and progression of PanCa [10]. However, among all tubulins, βIII and βIV isotypes play major role in PanCa progression, metastasis and chemoresistance [32]. Therefore, selective targeting of βIII and βIV-tubulins may improve the therapeutic response of PanCa. In this study, we have identified a novel βIII and βIV inhibitor (VERU-111), determined its therapeutic efficacy and investigated underlying cellular/molecular mechanisms using clinically relevant cell lines and preclinical mice PanCa models. Our results demonstrate that VERU-111 can effectively inhibit the growth of highly aggressive PanCa cells. To determine the specificity of β-tubulins targeting, we evaluated effect of VERU-111 on the expression of all β-tubulin isotypes by qPCR and Western blot analysis. These analyses suggest predominant inhibitory effect of this small molecule on the expression of βIII and βIV isotypes and differentially inhibition of (Fig. 2A-B), which are known to be involved in PanCa growth, metastasis and chemoresistance [44]. Our data also indicate that VERU-111 also differentially inhibits the expression of βI, and βVI isoforms. However, role of these tubulins in PanCa is not defined yet. Studies have also shown that the βIII tubulin expression level alters the sensitivity of microtubule destabilizing agents [45, 46]. Notably, available microtubule destabilizing agents such as vincristine, vinorelbine and nocodazole interact very weakly with βIII tubulin compared with other β-tubulin isotypes [47], which is clearly reflected in our comparative analysis of VERU-111 with colchicine, and vinorelbine. Our results demonstrated that VERU-111 is a more potent destabilizing agent of βIII/ βIV tubulin isotypes thus has profound therapeutic efficacy in PanCa models.

We also elucidated underlying cellular/molecular mechanisms of VERU-111 mediated targeting of PanCa associated β-tubulin isotypes (βIII/ βIV). It has been shown that βIII-tubulin is a direct target of miR-200c, thus its overexpression inhibits the metastatic phenotype of cancer cells and enhances sensitivity to chemotherapeutic agents [33]. Our results indicate that VERU-111 significantly (*p* < 0.01) restores the expression of miR-200c in PanCa cells/tumors, while miR-200c inhibitor diminish the effect of VERU-111 on the expression of β-III tubulin. These results strongly suggest that VERU-111 targets βIII-tubulin via replenishment of the expression of miR-200c. The invasive and migratory characteristics of cancer cells represent their metastatic phenotypes, which is an important step in cancer metastasis [48]. It has been shown that the overexpression of β-tubulins, specifically βIII and βIV, are involved in enhancing the metastatic potential of PanCa cells [44]. Our functional assays indicate that VERU-111 effectively inhibits invasive and migratory potential of PanCa cells. The effect of VERU-111 on cell cycle analysis was evaluated as it has been well documented that tubulin targeting drugs arrest cell cycle in the G2/M phase. G2 arrest of cell cycle prevent cancer cells from repairing DNA damage, forcing them into M phase; thus, the G2/M checkpoint is an ideal therapeutic target for anti-cancer drugs [49]. Our result indicates that VERU-111 arrested the cell cycle in the G2/M phase, which is in accordance with other tubulin targeting agents [49]. It has been reported that different class of cyclins and their cyclin-dependent kinases such as cyclin B1, Cdc2 and Cdc25C are involved in cell cycle progression [50, 51]. The inhibitory effect of VERU-111 on the expression of cyclin B1, Cdc2 and Cdc25C kinases explained its putative molecular effects on the cell cycle. Accumulating evidence suggests that G2/M cell cycle arrest leads to the induction of apoptosis, a mechanism of cell death. Our flow cytometry data indicate enhanced apoptosis in PanCa cells with the treatment of VERU-111. Bcl2 is an upstream effector molecule in the apoptotic pathway and has been recognized as a potent inhibitor of apoptosis and shown to be overexpressed in various types of cancer including PanCa [32, 52]. It forms a heterodimer with the apoptotic protein Bax, thereby neutralizing its apoptotic effects. Therefore, alteration in the ratio of Bax/Bcl2 is a crucial factor that plays an important role to determine whether cells will undergo apoptosis. Our results indicate that VERU-111 induces apoptosis via modulating mitochondrial proteins (Bcl2, Bcl-xL, Bax and Bad), activation of caspase 3 and 9 and PARP protein cleavage suggesting the involvement of an intrinsic apoptotic pathway. Our results also demonstrate that Z-VAD-FMK treatment rescue the PanCa cells from VERU-111 induce apoptosis. These results clearly suggest involvement of caspase 3 and 9 in VERU-111 induced apoptosis of PanCa cells. Our xenograft mouse model studies demonstrate that VERU-111 (50 μg/mice) effectively inhibits tumor growth with concomitant inhibition of βI, βIII and βIV tubulins and restoring expression of miR-200c. These results indicate that VERU-111 inhibits pancreatic tumor growth via inducing cell cycle arrest, apoptosis and restoration of miR-200c in PanCa cells. These results strongly suggest potent therapeutic efficacy of VERU-111 for PanCa treatment and further studies are warranted to translate this novel molecule for clinical use in future.

## Conclusion

Specific inhibitors of βIII and βIV isoforms could be an ideal candidate for the management of PanCa and overcoming chemo-resistance. We for the first time, demonstrate that VERU-111 is a novel synthetic compound, which preferentially targets βIII and βIV tubulin isotypes via restoration of miR-200c in PanCa cells. Overall, this study suggests that VERU-111 is a novel promising small molecule inhibitor of βIII and βIV tubulin isotypes, which could be useful as a monotherapy or in combination with conventional therapeutic regimens for the treatment of PanCa.

## Additional files

Additional file 1: **Figure S1.** (A) Synthesis and chemical structure of VERU-111. (B) ^1^H NMR characterization of VERU-111 using CD_2_Cl_2_ as the NMR solvent and full assignment of proton chemical shifts of VERU-111 (EtOAC: ethyl acetate). Spectrum was acquired on a Bruker Advance III 400 MHz NMR spectrometer equipped with a BBO probe at room temperature. (TIF 1059 kb)

Additional file 2: **Table S1.** List of Human β-Tubulin Isotypes qRT- PCR Primers used in this study. ^a^In a number of locations Inosine is inserted to break up runs of four or more Gs that might lead to unusually stable secondary structure.

Additional file 3: **Figure S3.** Effect of VERU-111 on invasion and migration of HPAF-II cells (A) Effect of VERU-111 on migration of HPAF-II cells as determined by scratch wound assay. Briefly, cells were seeded into 12-well plates and cultured up to 90% confluency. Cell scratch wound line in each well was generated using 200 μl pipette tip. The wounded cells monolayer were treated with indicated concentrations of VERU-111 for 24 h. Representative images (10x magnification) of HPAF-II cells were captured by phase contrast microscope at 0 and 24 h. (B) Effect of VERU-111 on migration of HPAF-II cells using 96-transwell chamber plate. Representative images of migratory HPAF-II cells of control and VERU-111 treatment groups after 24 h (i). Bar graphs (ii) indicating number of migratory HPAF-II cells in control and VERU-111 treatment groups. (C) Effect of VERU-111 on invasion of HPAF-II cells (i) as determined by Matrigel Invasion assay. Representative images of control and VERU-111 treatment groups were captured at 10x magnification after 24 h. Bar graphs (ii) indicate number of invaded HPAF-II cells. Results are presented as means ± SEM of three independent experiments. Asterisk (*) denotes the significant value *P* < 0.05. (TIF 3005 kb)

Additional file 4: **Figure S4.** Effect of VERU-111 on cells cycle distribution. (A) Effect of VERU-111 on cell cycle distribution of Panc-1 and AsPC-1 cells. Briefly, cells were treated with VERU-111 for 24 h. Cells in different phase was analyzed by flow cytometric analysis using Propidium Iodide. Representative images of histogram showing cell cycle distribution at different phases in Panc-1 (i) and AsPC-1(ii) cells. (TIF 1759 kb)

Additional file 5: **Figure S5.** Effect of VERU-111 on apoptosis induction in PanCa. (A) Effect of VERU-111 on apoptosis induction of Panc-1 and AsPC-1 cells. Briefly, cells were treated with indicated concentrations of VERU-111 for 24 h and apoptosis induction was analyzed by flow cytometry using Annexin V-7AAD Apoptosis kit. Data was acquired by using the Bio-RAD ZE5/Evererst Software v2.1 and analyzed using FlowJo v.10.3. (B) Effect of VERU-111 on mitochondrial membrane potential (ΔΨm) in Panc-1 and AsPC-1 cells as determined by TMRE staining. Representative images from three independent experiments are showing dose-dependent decrease of TMRE staining in Panc-1 and AsPC-1 cells (i). Bar graph showing dose-dependent decrease of ΔΨm as determined by quantitative analysis of TMRE staining by flow cytometry in Panc-10 and AsPC-1 (ii). Data represented as mean ± SEM of 3 independent experiments. Asterisk (*) denotes the significant value *p* < 0.05. C. Effect of VERU-111 alone or in combination with Z-VAD-FMK on apoptosis of PanCa. The cells were pretreated with Z-VAD-VAD-FMK for 2 h followed by VERU-111 (20 μM) for 24 h and apoptosis induction was analyzed by flow cytometry using Annexin V-7AAD Apoptosis kit. Representative images of histogram showing increase of apoptotic cells and data was acquired by using the Bio-RAD ZE5/Evererst Software v2.1 and analyzed using FlowJo v.10.3. (D) Quantitation of Western blots indicated in Fig. 5 E and F. The density ratio of pro-caspase-3 and 9, cleaved caspase-3 and 9 and PARP cleavage treated with different concentrations of VERU-111 (i) and general caspase inhibitor Z-VAD-FMK (20 μM for 2 h) followed by VERU-111 (20 nM) treatment for 24 h in PanCa cells (ii). Values are expressed as means ± SD. Experiments were repeated 3 times. Asterisk (*) denotes the significant value *P* < 0.05. (TIF 1351 kb)

Additional file 6: **Figure S6.** Effect of VERU-111 on weight of mice. AsPC-1 cells (2 × 10^6^ cells) were injected subcutaneously on the dorsal flanks of each mice. Mice were administered with VERU-111 (50 μg/mouse/week for three weeks i.e. 3 times per week for 3 weeks). Control group mice were administered with vehicle. Body weight of both the groups’ mice was recorded once in a week. Line graph representing constant increase in body weight of both the groups’s mice. Data represent mean ± SD value of *n* = 6 mice in each group. (TIF 464 kb)

Additional file 7: **Figure S2.** Western blot internal control (GAPDH) of various β-tubulin isotypes treated with VERU-111 in PanCa cells. Panc-1 (i) and AsPC-1(ii) cells. Were treated with vehicle or indicated concentrations of VERU-111 for 24 h. Cell lysates were prepared and 40 μg protein was subjected for Western blot analysis. Equal loading of protein in each well was confirmed by stripping and re-probing the blots with GAPDH. Each experiment was repeated two times and similar results were obtained. (TIF 1222 kb)

## Abbreviations

FBS: Fetal bovine serum
FDA: Food and Drug Administration
IHC: Immunohistochemistry
ISH: In situ hybridization
PanCa: Pancreatic Cancer
PBS: Phosphate saline buffer
PI: Propidium iodide
PMSF: Phenylmethanesulfonyl fluoride
qRT-PCR: Quantitative-Reverse Transcription Polymerase Chain Reaction
TMRE: tetramethylrhodamine
UTHSC-IACUC: UTHSC Institutional Animal Care and Use Committee and
