# Supplementary figures and images for: Therapeutic efficacy of a novel βIII/βIV-tubulin inhibitor (VERU-111) in pancreatic cancer
Source: J Exp Clin Cancer Res. 2019 Jan 23;38:29. doi: 10.1186/s13046-018-1009-7 (PMC6343279; doi:10.1186/s13046-018-1009-7)

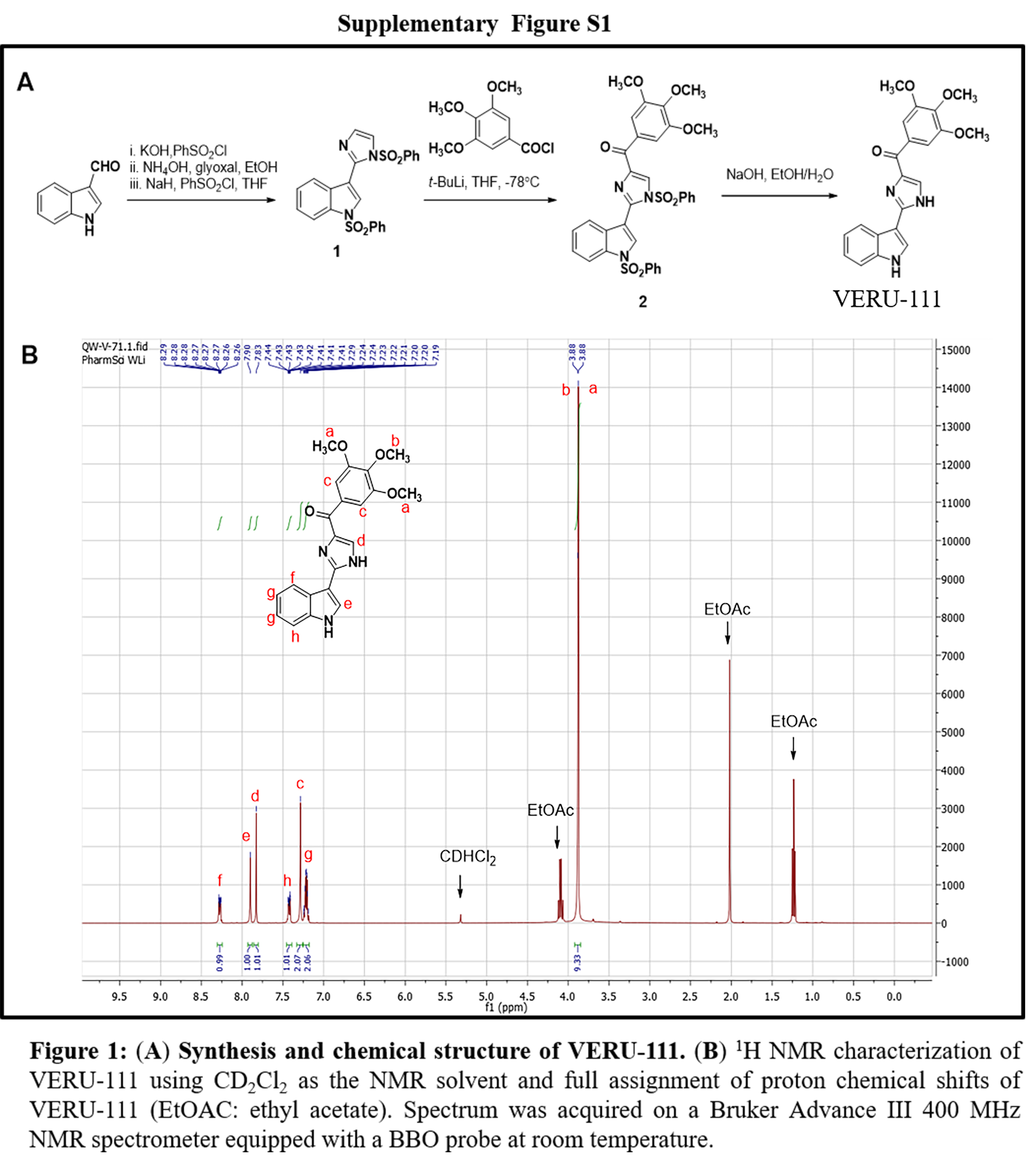

Supplement: Supplementary file 1 — Figure S1. (A) Synthesis and chemical structure of VERU-111. (B) 1H NMR characterization of VERU-111 using CD2Cl2 as the NMR solvent and full assignment of proton chemical shifts of VERU-111 (EtOAC: ethyl acetate). Spectrum was acquired on a Bruker Advance III 400 MHz NMR spectrometer equipped with a BBO probe at room temperature. (TIF 1059 kb) [file 13046_2018_1009_MOESM1_ESM.tif]

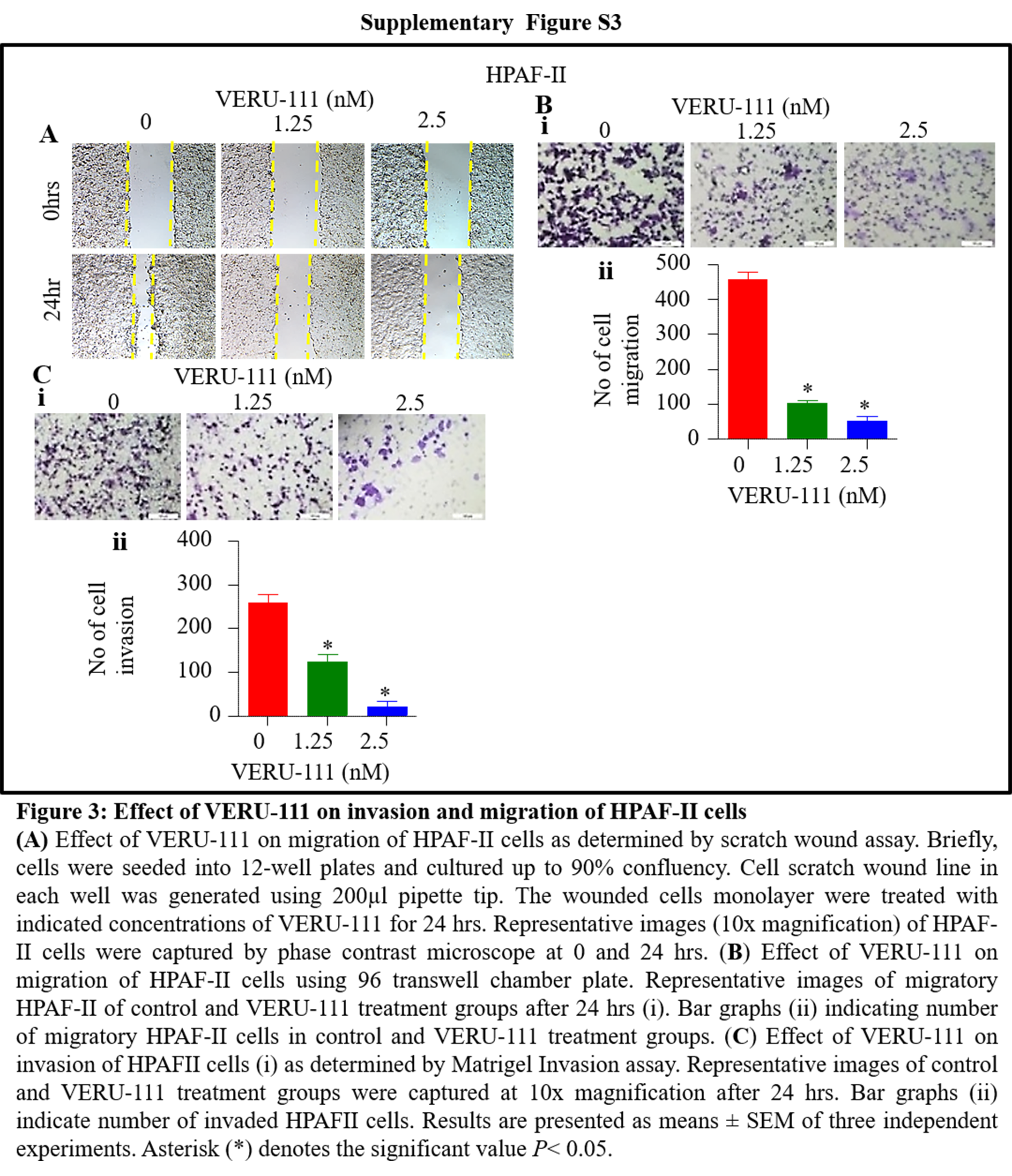

Supplement: Supplementary file 3 — Figure S3. Effect of VERU-111 on invasion and migration of HPAF-II cells (A) Effect of VERU-111 on migration of HPAF-II cells as determined by scratch wound assay. Briefly, cells were seeded into 12-well plates and cultured up to 90% confluency. Cell scratch wound line in each well was generated using 200 μl pipette tip. The wounded cells monolayer were treated with indicated concentrations of VERU-111 for 24 h. Representative images (10x magnification) of HPAF-II cells were captured by phase contrast microscope at 0 and 24 h. (B) Effect of VERU-111 on migration of HPAF-II cells using 96-transwell chamber plate. Representative images of migratory HPAF-II cells of control and VERU-111 treatment groups after 24 h (i). Bar graphs (ii) indicating number of migratory HPAF-II cells in control and VERU-111 treatment groups. (C) Effect of VERU-111 on invasion of HPAF-II cells (i) as determined by Matrigel Invasion assay. Representative images of control and VERU-111 treatment groups were captured at 10x magnification after 24 h. Bar graphs (ii) indicate number of invaded HPAF-II cells. Results are presented as means ± SEM of three independent experiments. Asterisk (*) denotes the significant value P < 0.05. (TIF 3005 kb) [file 13046_2018_1009_MOESM3_ESM.tif]

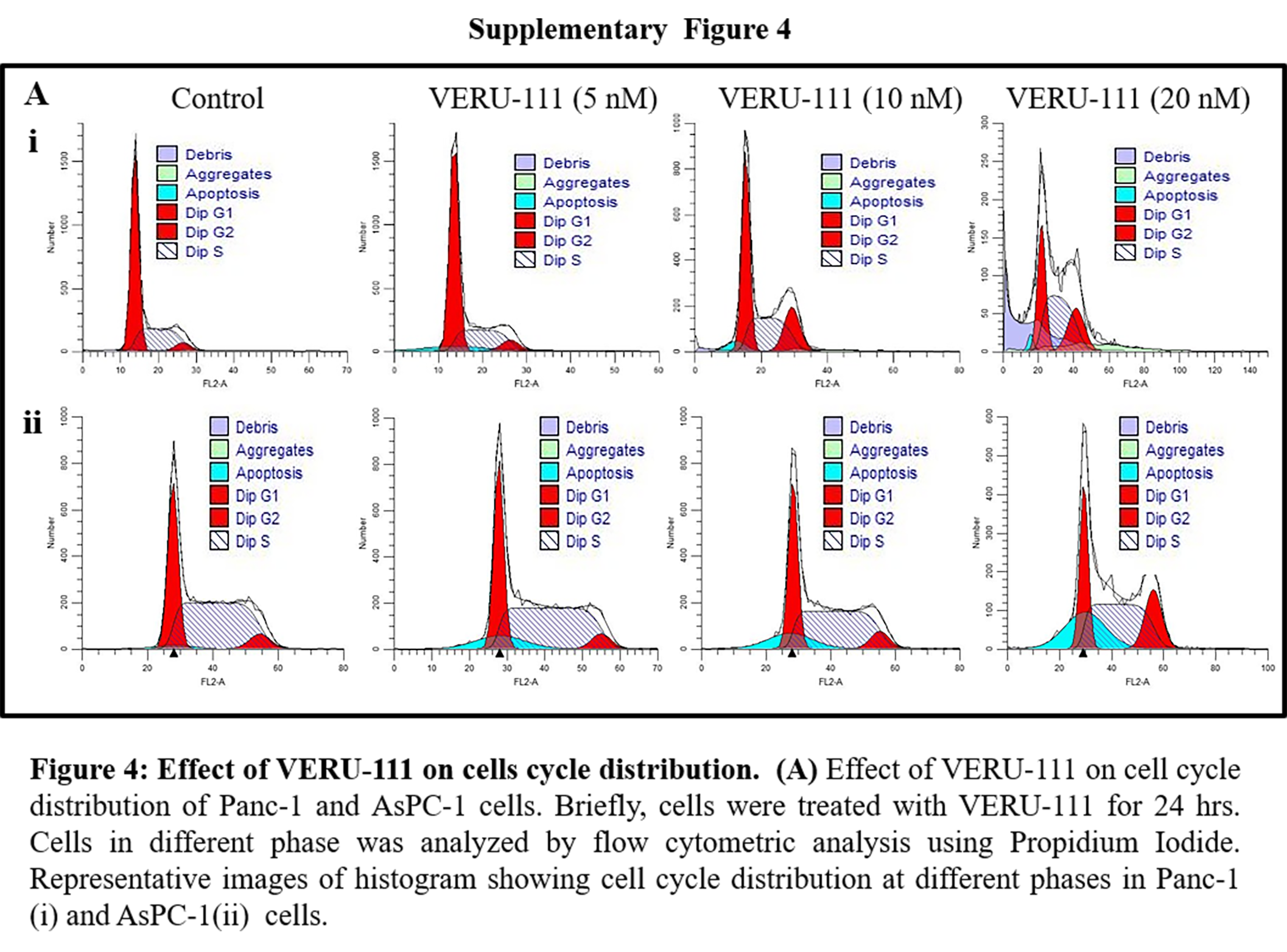

Supplement: Supplementary file 4 — Figure S4. Effect of VERU-111 on cells cycle distribution. (A) Effect of VERU-111 on cell cycle distribution of Panc-1 and AsPC-1 cells. Briefly, cells were treated with VERU-111 for 24 h. Cells in different phase was analyzed by flow cytometric analysis using Propidium Iodide. Representative images of histogram showing cell cycle distribution at different phases in Panc-1 (i) and AsPC-1(ii) cells. (TIF 1759 kb) [file 13046_2018_1009_MOESM4_ESM.tif]

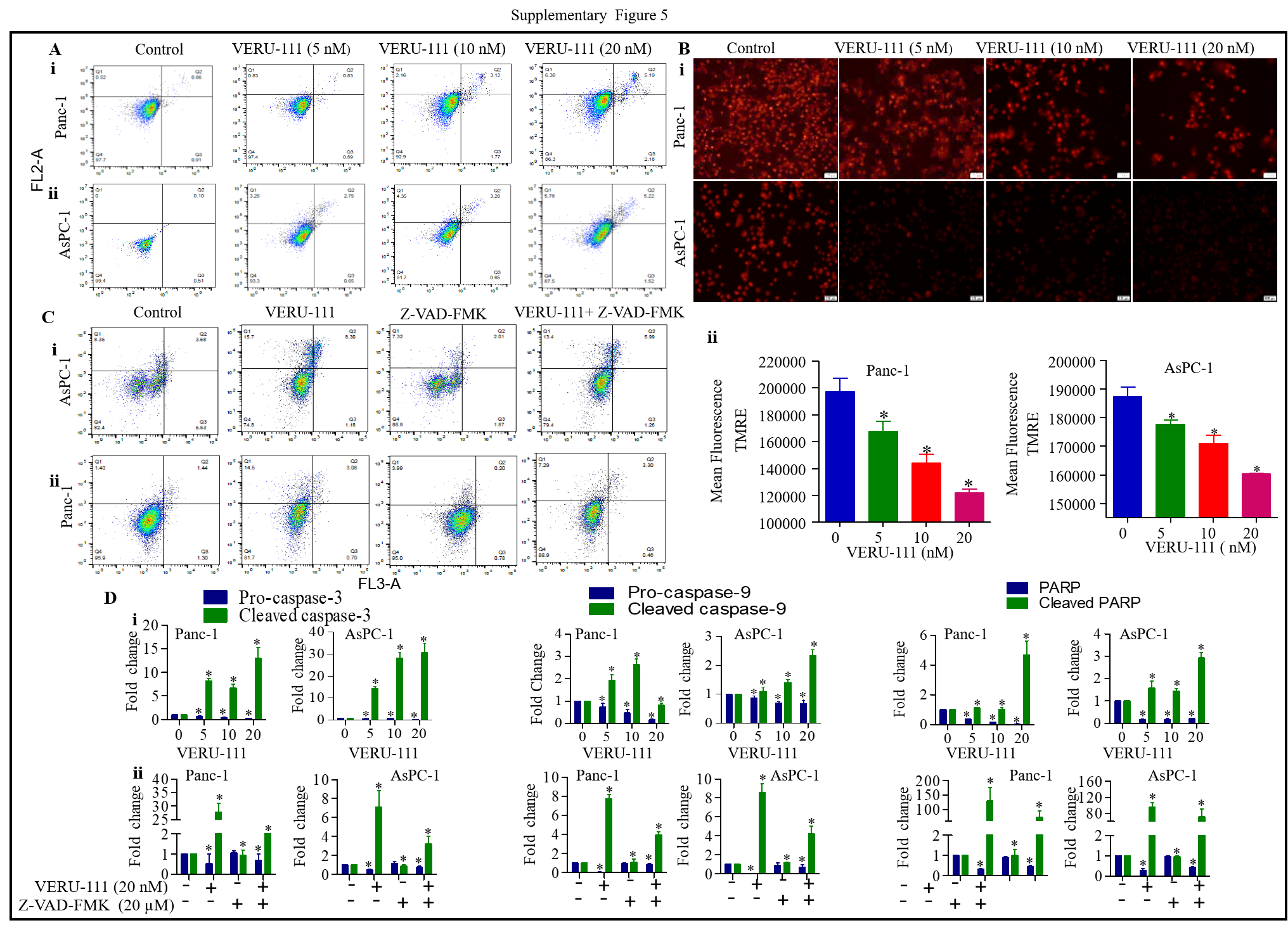

Supplement: Supplementary file 5 — Figure S5. Effect of VERU-111 on apoptosis induction in PanCa. (A) Effect of VERU-111 on apoptosis induction of Panc-1 and AsPC-1 cells. Briefly, cells were treated with indicated concentrations of VERU-111 for 24 h and apoptosis induction was analyzed by flow cytometry using Annexin V-7AAD Apoptosis kit. Data was acquired by using the Bio-RAD ZE5/Evererst Software v2.1 and analyzed using FlowJo v.10.3. (B) Effect of VERU-111 on mitochondrial membrane potential (ΔΨm) in Panc-1 and AsPC-1 cells as determined by TMRE staining. Representative images from three independent experiments are showing dose-dependent decrease of TMRE staining in Panc-1 and AsPC-1 cells (i). Bar graph showing dose-dependent decrease of ΔΨm as determined by quantitative analysis of TMRE staining by flow cytometry in Panc-10 and AsPC-1 (ii). Data represented as mean ± SEM of 3 independent experiments. Asterisk (*) denotes the significant value p < 0.05. C. Effect of VERU-111 alone or in combination with Z-VAD-FMK on apoptosis of PanCa. The cells were pretreated with Z-VAD-VAD-FMK for 2 h followed by VERU-111 (20 μM) for 24 h and apoptosis induction was analyzed by flow cytometry using Annexin V-7AAD Apoptosis kit. Representative images of histogram showing increase of apoptotic cells and data was acquired by using the Bio-RAD ZE5/Evererst Software v2.1 and analyzed using FlowJo v.10.3. (D) Quantitation of Western blots indicated in Fig. 5 E and F. The density ratio of pro-caspase-3 and 9, cleaved caspase-3 and 9 and PARP cleavage treated with different concentrations of VERU-111 (i) and general caspase inhibitor Z-VAD-FMK (20 μM for 2 h) followed by VERU-111 (20 nM) treatment for 24 h in PanCa cells (ii). Values are expressed as means ± SD. Experiments were repeated 3 times. Asterisk (*) denotes the significant value P < 0.05. (TIF 1351 kb) [file 13046_2018_1009_MOESM5_ESM.tif]

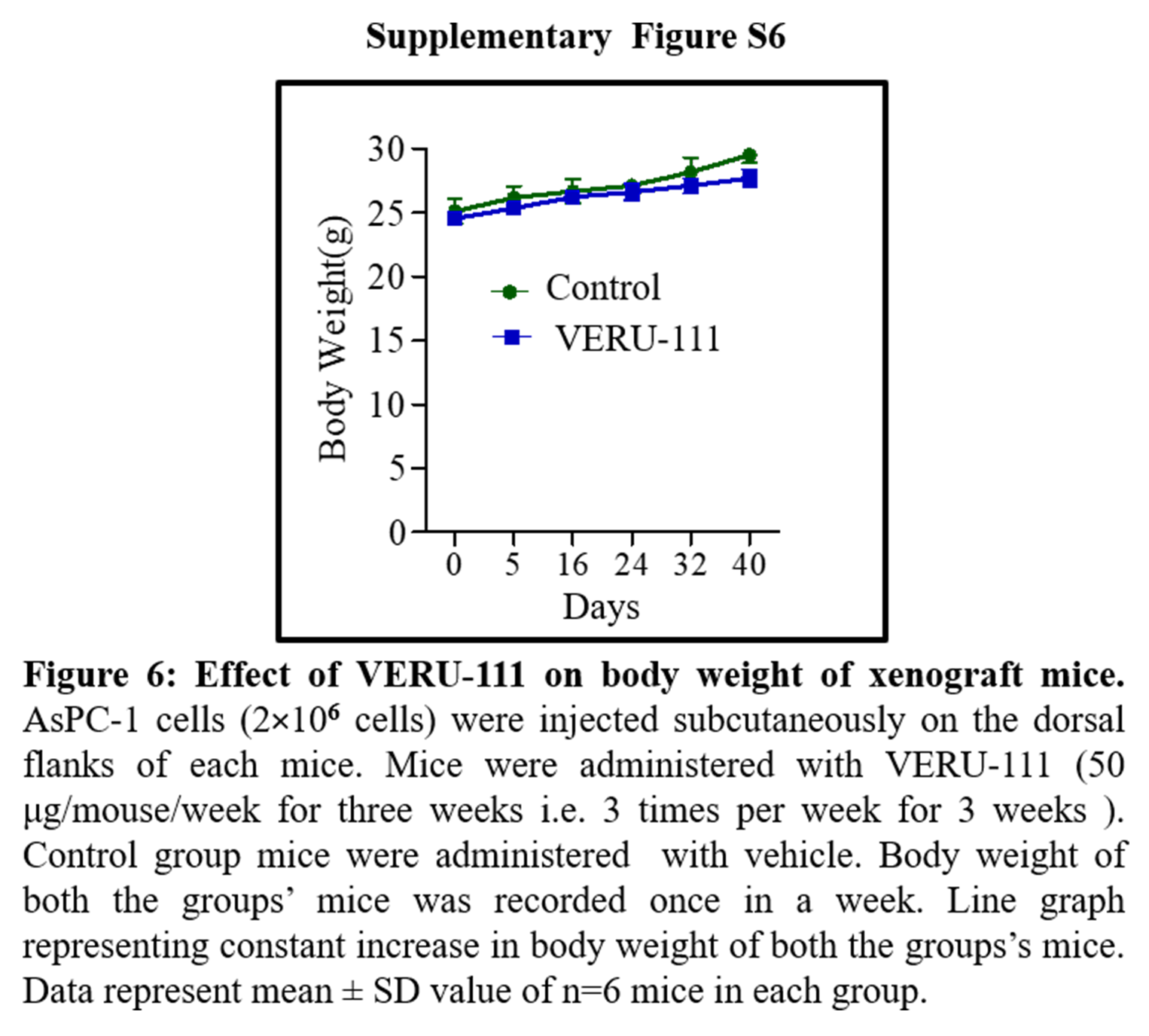

Supplement: Supplementary file 6 — Figure S6. Effect of VERU-111 on weight of mice. AsPC-1 cells (2 × 10^6^ cells) were injected subcutaneously on the dorsal flanks of each mice. Mice were administered with VERU-111 (50 μg/mouse/week for three weeks i.e. 3 times per week for 3 weeks). Control group mice were administered with vehicle. Body weight of both the groups’ mice was recorded once in a week. Line graph representing constant increase in body weight of both the groups’s mice. Data represent mean ± SD value of n = 6 mice in each group. (TIF 464 kb) [file 13046_2018_1009_MOESM6_ESM.tif]

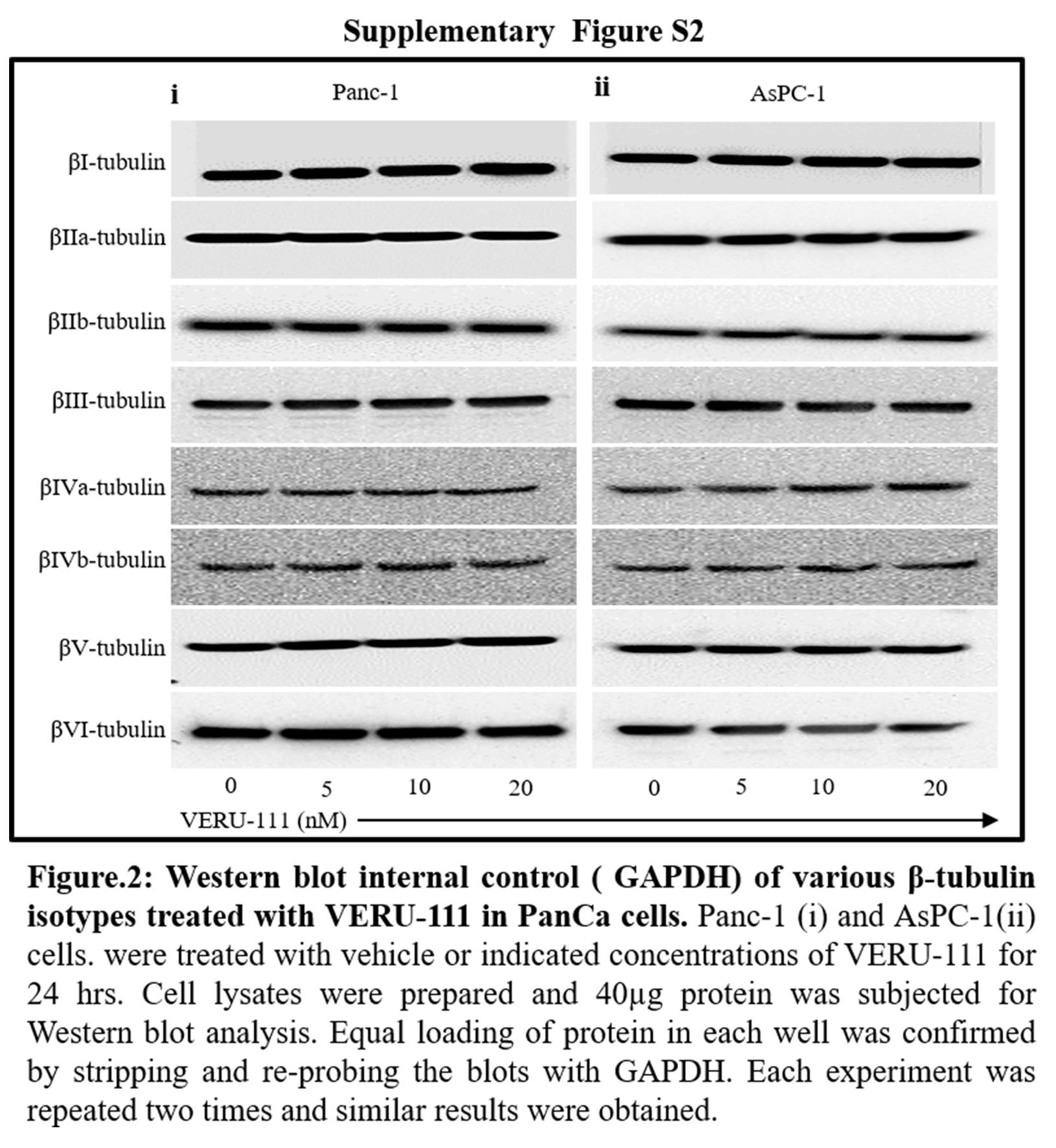

Supplement: Supplementary file 7 — Figure S2. Western blot internal control (GAPDH) of various β-tubulin isotypes treated with VERU-111 in PanCa cells. Panc-1 (i) and AsPC-1(ii) cells. Were treated with vehicle or indicated concentrations of VERU-111 for 24 h. Cell lysates were prepared and 40 μg protein was subjected for Western blot analysis. Equal loading of protein in each well was confirmed by stripping and re-probing the blots with GAPDH. Each experiment was repeated two times and similar results were obtained. (TIF 1222 kb) [file 13046_2018_1009_MOESM7_ESM.tif]
