# Supplementary material for: Therapeutic efficacy of a novel βIII/βIV-tubulin inhibitor (VERU-111) in pancreatic cancer
Source: J Exp Clin Cancer Res. 2019 Jan 23;38:29. doi: 10.1186/s13046-018-1009-7 (PMC6343279; doi:10.1186/s13046-018-1009-7)
Supplement: Supplementary file 2 — Table S1. List of Human β-Tubulin Isotypes qRT- PCR Primers used in this study. aIn a number of locations Inosine is inserted to break up runs of four or more Gs that might lead to unusually stable secondary structure. [file 13046_2018_1009_MOESM2_ESM.docx]

| Protein | Gene  name | Accession number | Forward sequence 5’–3’ | Reverse sequence 5’–3’ | Amplicon length (bp) |
| --- | --- | --- | --- | --- | --- |
| β-I | TUBB | NM_178014.2 | CCC CAT ACA TAC CTT GAG GCG A | GCC AAA AGG ACC TGA GCG AA | 290 |
| β-IIA | TUBB2A | NM_001069.2 | CTC AGA TCA ATC GTG CAT CCT TAG TGA ACT TCT GT | GTA TAG ATA CCT TCA CAG ACA ATA CTG TAA TTT TTA GAG GAG TTC CAC A | 167 |
| β-IIB | TUBB2B | NM_178012.4 | ACG GGT TAG GGA AAG CGG A | TTC CGA CAC AAA CGT TTA TGT GA | 242 |
| β-III | TUBB3 | NM_006086.2 | ATG AGG GAG ATC GTG CAC AT | CCC CTG AGC GGA CAC TGT | 238 |
| β-IVA | TUBB4 | NM_006087.2 | TCT CCG CCG CAT CTT CCA | GCT CTG GIaG ACA TAA TTT CCT CCT | 274 |
| β-IVB | TUBB2C | NM_006088.5 | GCT GTT TGT CTA CTT CCT CCT GCT | CAG TTG TTC CCA GCA CCA CTC T | 349 |
| β-V | TUBB6 | NM_032525.1 | CGG IaGA GGA AGC TTT TGA GGA T | CTG GGT AGA ACC CGC AAT TCT CT | 244 |
| β-VI | TUBB1 | NM_030773.2 | AGT TGT GTT GGG CTC ACA CCA | TTG CCA CAC TGG CCA ATC TGA | 133 |
| GAPDH | GAPDH | NM_002046.3 | GCC GAG CCA CAT CGC TCA GAC ACC A | GGG ATC TCG CTC CTG GAA GAT GGT GAT GGG A | 270 |

**Supplementary Table S1:** List of Human β-Tubulin Isotypes qRT- PCR Primers used in this study
^a^In a number of locations Inosine is inserted to break up runs of four or more Gs that might lead to unusually stable secondary structure.
